# Beyond Adding Years to Life: Health-related Quality-of-life and Functional Outcomes in Patients with Severe Aortic Valve Stenosis at High Surgical Risk Undergoing Transcatheter Aortic Valve Replacement

**DOI:** 10.2174/1573403X09666131202121750

**Published:** 2013-11

**Authors:** Marcus-André Deutsch, Sabine Bleiziffer, Yacine Elhmidi, Nicolo Piazza, Bernhard Voss, Ruediger Lange, Markus Krane

**Affiliations:** Department of Cardiovascular Surgery, German Heart Center Munich, Technische Universität München, Germany

**Keywords:** Aortic valve stenosis, transcatheter aortic valve implantation, functional outcomes, health-related quality-of-life.

## Abstract

Aortic valve stenosis (AVS) is the most frequent acquired valvular heart disease in western industrialized countries and its prevalence considerably increases with age. Once becoming symptomatic severe AVS has a very poor prognosis. Progressive and rapid symptom deterioration leads to an impairment of functional status and compromised healthrelated quality-of-life (HrQoL) simultaneously. Until recently, surgical aortic valve replacement (SAVR) has been the only effective treatment option for improving symptoms and prolonging survival. Transcatheter aortic valve replacement (TAVR) emerged as an alternative treatment modality for those patients with severe symptomatic AVS in whom the risk for SAVR is considered prohibitive or too high. TAVR has gained clinical acceptance with almost startling rapidity and has even quickly become the standard of care for the treatment of appropriately selected individuals with inoperable AVS during recent years. Typically, patients currently referred for and treated by TAVR are elderly with a concomitant variable spectrum of multiple comorbidities, disabilities and limited life expectancy. Beyond mortality and morbidity, the assessment of HrQoL is of paramount importance not only to guide patient-centered clinical decision-making but also to judge this new treatment modality. As per current evidence, TAVR significantly improves HrQoL in high-surgical risk patients with severe AVS with sustained effects up to two years when compared with optimal medical care and demonstrates comparable benefits relative to SAVR.

Along with a provision of a detailed overview of the current literature regarding functional and HrQoL outcomes in patients undergoing TAVR, this review article addresses specific considerations of the HrQoL aspect in the elderly patient and finally outlines the implications of HrQoL outcomes for medico-economic deliberations.

## 1. INTRODUCTION

Aortic valve stenosis (AVS) is currently the most frequent acquired native valve disease in western industrialized countries and its prevalence considerably increases with age. Results from the Cardiovascular Health Study unveiled that in patients aged 65-75 years, 75-85 years, and older than 85 years severe AVS is present in 1.3%, 2.4%, and 4%, respectively. Symptoms of AVS are latent until there is critical narrowing of the aortic valve resulting in left ventricular hypertrophy, increased left ventricular diastolic pressure and increased myocardial oxygen demand causing subendocardial ischemia. Once cardinal symptoms - angina pectoris, syncope, dyspnea and heart failure - develop in the course of the disease, prognosis is dismal. Mortality of medically-treated severe, symptomatic AVS carries a high mortality achieving rates of about 25% per year [1]. Results from the PARTNER (Placement of AoRtic TraNscathetER Valve) trial showed an even poorer prognosis for elderly high-surgical risk patients who were treated medically: survival at 1 year was only 50% [2]. Additionally, the disease burden of symptomatic AVS is associated with reduced activity levels until quality of life suffers from an inability to participate in the daily activities that make life meaningful. According to well established consensus guidelines, surgical aortic valve replacement (SAVR) is the treatment of choice for high-grade, symptomatic AVS with no explicit restrictions for surgical intervention related to advanced age per se [3, 4]. In patients selected for isolated SAVR, the perioperative risk is low and outcomes have continued to improve due to refinements of operative techniques, advances in cardiopulmonary bypass and perioperative care. Overall operative mortality rates for isolated SAVR range from 2.5 to 4.0% in younger patients. However, they tend to be higher in octogenarians and nonagenarians (4.9% to 9.6%). In patients presenting with extensive significant co-morbidities the risk of death (up to 25%) and morbidity as well as length of hospitalization is markedly increased [5-7]. For this reason the ACC/AHA and ESC guidelines acknowledge that special considerations are required in elderly patients with AVS, since age-related and comorbid conditions are more common among higher age strata [3, 4]. 

With increasing longevity and high prevalence of AVS in the elderly, surgeons and cardiologists will be more frequently confronted with difficult treatment algorithms. Owing to advanced age and the presence of significant extracardiac co-morbidity, a considerable number of patients are therefore considered ineligible for SAVR because operative risk is projected to be unacceptably high. It has been estimated that approximately 30-48% of high risk elderly patients are denied SAVR [8-10]. While allowing the implantation of a prosthetic heart valve without the need for sternotomy and the use of cardiopulmonary bypass, transcatheter aortic valve implantation (TAVR) has been developed as an alternative, less-invasive treatment modality for those patients in whom risk for SAVR is considered prohibitive or too high. As a relatively new procedure TAVR has gained clinical acceptance in almost startling rapidity since its first-in-man application in 2002. The reproducible safety and efficacy not only increased confidence in the technique but also established a novel, valuable treatment option in the sizeable group of symptomatic patients with severe AVS previously denied access to treatment. To date, more than 50,000 patients have been implanted worldwide with one of the two commercially approved TAVR devices, including the balloon-expandable Edwards SAPIEN Transcatheter Heart Valve (Edwards LifeSciences, Irvine, California) and the self-expanding CoreValve Revalving System (Medtronic, Minneapolis, Minnesota) and it has been demonstrated that good short- and mid- term results can be achieved. A number of additional transfemoral and transapical devices are under evaluation. Recently, multiple trails have reported robust favourable clinical outcomes at 2- and 3-year after TAVR [11-20].

Patients treated by TAVR are elderly and present with a variable spectrum of multiple co-morbidities and limited life expectancy. For these patients, the primary goal is not solely longevity, but rather are safety, survival, and the restoration of comfort in daily life. Important considerations include functional mobility, quality of life, and maintenance of their independent status [21]. Measures of morbidity and mortality do not provide complete information about physical, functional, emotional, and mental well-being and can be supplemented by patients’ perceptions of their recovery. Health-related quality-of-life (HrQoL) evaluation is becoming an increasingly important aspect of assessing the outcome of any therapeutic intervention. In clinical practice, the use of HrQoL measures is a way of focusing treatment on the patient rather than on the disease. Therefore, beyond the traditional outcome parameters mortality and morbidity, functional status as well as HrQoL dynamics has to be assessed not only to guide patient-centered clinical decision-making but also to judge this new treatment modality. Accordingly, in several position statement and consensus documents, the assessment of HrQoL was specified to be a clinical benefit endpoint of crucial importance for TAVR clinical trials [22-24]. As to that, several studies reporting on the short- and mid-term HrQoL outcomes in patients undergoing TAVR have been published recently. Especially with the availability of data from the randomized controlled US PARTNER trial Cohort A and B the knowledge gap is now closing. Along with a provision of a detailed overview of the current literature regarding the effects of TAVR on functional and quality-of-life outcomes, this review article addresses the specific considerations of the quality-of-life aspect in the elderly co-morbid patient presenting with severe AVS. Finally, the implications of HrQoL results on medico-economic deliberations and some important future directions are outlined.

## 2. SPECIFIC CONSIDERATIONS IN THE ELDERLY HIGH-RISK PATIENT REFERRED FOR TAVR: PATIENT SELECTION AND RATIONALE FOR HrQoL ASSESSMENT

Although indication and treatment algorithms for SAVR are outlined by well-defined guidelines, patient selection for TAVR is still one of the most challenging issues in clinical practice [2, 3]. 

In the aged and frail population currently presenting for TAVR, in which clinicians are frequently confronted with a previously understudied group of individuals. Variation of the functional status and physiological losses in combination with individual and variable pattern of chronic diseases is a hallmark among the older patient populations creating unique heterogeneity among elderly patients presenting with severe AVS [8, 25]. Typically, patients currently referred for and treated by TAVR present with a concomitant variable spectrum of significant multiple co-morbidities, disabilities and limited life expectancy. There is general agreement that patients with limited life expectancy due to advanced age, significant concomitant comorbid conditions such as porcelain aorta, malignancy, history of chest radiation, chronic respiratory or renal insufficiency, cirrhosis, pulmonary artery hypertension, right ventricular failure, history of prior cardiac surgery, among others, are not appropriate candidates for SAVR. Patients currently selected for TAVR are elderly (average age typically over 80 years), with symptomatic severe AVS (mean gradient 45> mmHg), significant comorbidities, and an average logistic EuroSCORE between >23% and >16% [12], indicating a significant risk with SAVR. Patients in the PARTNER trial Cohort B had a high frequency of coexisting conditions that contributed to the surgeons’ determination of inoperability, such as porcelain aorta (15.1%), chest-wall deformity or prior chest-wall irradiation (13.1%), oxygen-dependent respiratory insufficiency (23.5%), and frailty, according to prespecified criteria (23.1%). In the light of lacking guideline recommendations, the determination of inoperability in the individual patient still largely depends on the judgment of the treating medical team [24]. Frailty and related conditions of debility and deconditioning are known to impair recovery from major heart surgery despite operative survival and hospital discharge [26]. On the other hand, all the SAVR precluding factors add complexity to any invasive intervention and by adding an additional burden on patients during recovery finally affect the extent of quality-of-life benefits that can be expected after the intervention hinder improvement in health status. STS risk score and EuroSCORE give useful information concerning mortality risks, but they are not able to predict symptom resolution, quality-of-life improvement, or return to independent living. This renders the question whether the benefit of surgery outweighs the risk of intervention and the decision between continued medical management and surgical intervention, a complex dilemma. 

Essentially, procedural success of any new treatment modality is judged by answering two questions: Does the intervention prolong life and, does the intervention relieve symptoms and suffering at acceptable risks? 

For geriatric patients electing to undergo cardiac surgery, the primary goal is not just solely the prolongation of live but rather the restoration of the comfort in daily life which is not only to return the patient to some status quo ante, but a gain or at least the full restoration of a satisfactory quality of life comparable to a level before onset and progression of limiting symptoms [27]. During the process of deciding whether to offer cardiac surgical intervention to elderly patients, the relief of symptoms and improvement in quality of life should assume more importance than the issue of increased life expectancy. Quality of life is therefore a key patient-centered outcome. Although death is the lowest possible functional status, for many, survival marked by reduced physical function or independence may be worse than death. Therefore, even though TAVR would be technically feasible, if the procedure merely prolonged a miserable existence, it would not be very beneficial to patients. Thus, it is of particular interest in this high-risk patient population selected for TAVR in whom significant co-morbidities provided the rational for refusal to be accepted for SAVR/for considering the patient inoperable, to evaluate whether despite the presence of such co-morbidities, whether quality of life can still be restored or even improved when the additive effect of severe aortic stenosis and related sequela are rectified [28]. The traditional outcome parameters, though crucial, may fail to appreciate the full effects on emotional, physical, functional and mental well-being, and provide only very limited information about the patients’ postoperative physical, functional and emotional recovery, i.e. improvements in haemodynamic performance may not necessarily manifest itself in changes of comfort in daily activities. 

The relevant key questions to be answered in this respect are as follows: Firstly, does TAVR, despite the presence of co-morbidities, restore or improved when the additive effect of severe aortic stenosis and related sequela are rectified when compared to “no-option” patients optimal medical care or patients to patients at high surgical risk undergoing SAVR. Secondly, are these benefits durable in the longer term. Thirdly, are there independent prognostic indicators for the extent or absence of HrQoL benefits that can be identified to guide better risk stratification, patient selection and patient-centered clinical decision making. Fourthly, how relevant postprocedural complications such as paravalvular aortic regurgitation, need for pacemaker implantation and stroke are influence HrQoL and finally, based on HrQoL outcomes, is TAVR a cost-effective treatment modality.

## 3. HEALTH-RELATED QUALITY-OF-LIFE: DEFINITION AND EVALUATION TOOLS USED IN TAVR-RELATED STUDIES 

Elkinton in 1966 described quality of life as ‘not just the absence of death but life with the vibrant quality that was associate with the vigour of youth’ [29]. There is no universal agreement on the definition of ‘quality of life’. Quality of life is a key patient-centered outcome. As applied to medicine it is more specifically known as ‘health-related quality of life’ or ‘subjective health status’. HrQoL assessments provide additional information on patient status and postprocedural recovery thereby extending an outcome assessment process beyond the conventional clinical outcome measures. HrQoL assessments have been described to be of value for both risk assessment and as an outcome measure [30]. HrQoL data is collected and quantitated using structured questionnaires, usually consisting of a number of items (questions or statements) which tap various dimensions of quality of life. 

Assessment instruments are multidimensional and designed to objectify a patient’s subjective health perception integrating not only functional and physical dimensions of the disease, but also the psychologic and social dimensions. In this way, qualitative information is converted into quantitative data and scores given to every dimension represented by the questionnaire. Several HrQoL patient-reported outcome measure assessment tools have been used in TAVR-related studies: the Medical Outcomes Trust Short Form 36-Item Health Survey (SF-36) and the Short-Form SF-12, the Minnesota living with Heart Failure questionnaire (MLHFQ), the Kansas City Cardiomyopathy Questionnaire (KCCQ) and the EuroQoL 5 (EQ-5D). 

**Fig. (1):**
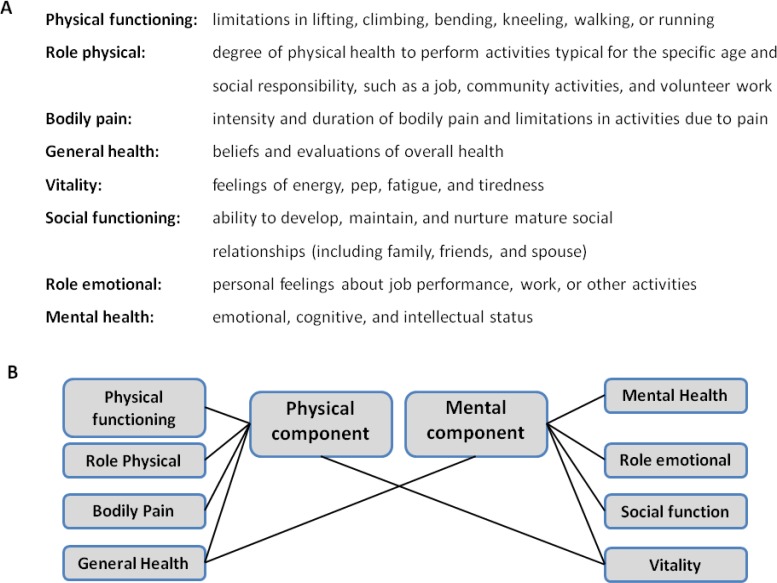
Domains of the SF-36/SF-12 health survey questionnaire.

The SF-36 short-form health survey is the worlds most extensively used multipurpose instrument demonstrating a strong support for validity and reliability which has been validated by a myriad of various disease conditions and a whole spectrum of different age groups [31]. The SF-36 assay is a generic measure, as opposed to others that target a specific age, disease or treatment group. It is a self-administered instrument which takes about 15 minutes to complete. With a 36-item questionnaire the SF-36 yields an 8-scale profile of eight general health parameters reflecting functional health and well-being scores (physical functioning, role-physical, bodily pain, general health, role-emotional, social functioning, vitality and mental health). The number of possible responses per item varies from 2 to 6. For each parameter, scores are calculated and transformed to a scale from 0 to 100, with higher scores reflecting a better HrQoL [32]. Each item measures functioning in different aspects of daily life (see Fig. **1A**). The 8 parameters are summarized in two meta-scores: the physical (PCS) and the mental component summary score (MCS) (see Fig. **1B**). The SF-12 is a shortened and simplified tool which was derived from the longer Short Form-36 questionnaire. PCS and MCS obtained from the SF-12 correlate highly with those calculated using the original longer questionnaire. The majority of studies evaluating HRQoL after TAVR have used the SF-12 questionnaire because of its brevity. The SF-12 has been reported to have considerably lower rate of floor and ceiling effects; however, this may be at the cost of losing detailed information about separate health-status domains [33].

The content of the Minnesota Living with Heart Failure questionnaire (MLHFQ) is a 21-item structured questionnaire and was designed to measure the disease-specific effects of heart failure and its treatment on individual patient´s key physical, emotional, social and mental dimensions of quality of life. The questionnaire can be self-administered or applied in a 5-minute interview. It is evaluated using a 6-point Likert scale ranging from 0 (no impact/not applicable (best score) to 5 (severe impact (worst score)). Physical and emotional dimension scores (ranges: 0 to 40 (8 items) and 0 to 25 (5 items), respectively. Summation of the responses yields a total MLHFQ score for each patient ranging between 0 and 105, whereas lower scores indicate better HrQoL [34, 35]. 

The Kansas City Cardiomyopathy Questionnaire (KCCQ) is a 23-item questionnaire designed and validated for the evaluation of self-reported disease-specific health status in patients with heart failure. The conceptual domains include symptoms, physical limitation, social limitation, self-efficacy, and quality-of-life. Individual scales and overall summary score range from 0 to 100, whereas higher scores indicate fewer symptoms and better QOL. The KCCQ summary scores have previously been reported to correlate with New NYHA class and has been shown to independently predict mortality and health care costs in heart failure populations [36, 37].

The EQ-5D is a 5-domain generic health state classification system reflecting mobility, self-care, usual activities, pain/discomfort, anxiety/depression. It is cognitively simple, taking only a few minutes to complete. The health states defined by the EQ-5D have been transformed to preference-based utilities based on responses from a US reference population. Values range from 0 to 1, whereas 1 represents ideal health and 0 represens the worst health state (usually death). Because of its low sensitivity, EQ-5D should be used as a supplementary tool and not as a substitute for other instruments [38].

The question at the end of the day is: regardless of statistical significances, what represents a clinically meaningful change in all these HrQoL metrics? Clinically relevant change in the SF PCS were reported to be one-half of one standard deviation of the mean composite score which is approximately equivalent to 4 to 7 points 9,10. Minimum clinically important differences on the SF-12 summary scales are 2 to 2.5 points [39, 40]. A change ≥5 points in total score has been considered as clinically meaningful for the MLHFQ [34]. Established thresholds for clinically relevant changes in the KCCQ are: dead; worse (decrease of >5 points from baseline); unchanged (change between 5 and 5 points); slightly improved (increase between 5 and 10 points); moderately improved (increase between 10 and 20 points); and substantially improved (increase >20 points) [37].

## 4. SUMMARY OF HrQoL AND FUNCTIONAL OUTCOMES IN PATIENTS UNDERGOING TAVR

After several registries have conclusively demonstrated safety, efficacy with good clinical results in the short- and long-term, evidence is currently accumulating that TAVR in high surgical risk patients with severe symptomatic AVS is associated with marked functional and HrQoL benefits. Significant HrQoL improvements are detectable as early as 1 month post TAVR followed by clinical stabilisation and detectable up to one year. The 1-year health status of TAVR population has been consistently shown to become similar to age-matched general population norms. Table **1** summarizes the design and reported outcomes of TAVR- related studies published so far evaluating HrQoL.

### HrQoL Results from the Prospective US-PARTNER Trial/Cohort A and B

**Fig. (2):**
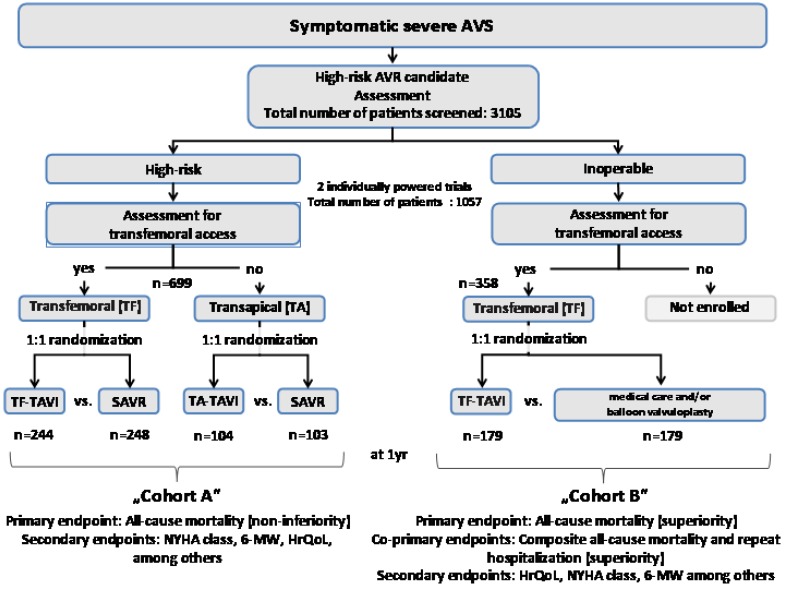
Study design of the US PARTNER randomized controlled trial.

The Placement of AoRTic TraNscathetER Valve trial in the U.S. (US PARTNER; ClinicalTrials.gov identifier NCT00530894) was a multicenter pivotal study and incorporated two parallel prospective, multicenter, randomized, active-treatment-controlled clinical trials evaluating the safety and effectiveness of the Edwards Sapien THV transcatheter aortic valve. Being the first and only randomized controlled clinical trial with the largest patient population followed-up and described so far, this study provided very important insights into the HrQoL status and changes in this specific patient population in comparison with a medical treatment group. Patients who were considered high surgical risk, eligible for transfemoral (TF) access were stratified into Cohort A and randomized to TF-TAVR or SAVR (control). Cohort A patients ineligible for TF access were evaluated as candidates for transapical (TA) delivery and, if appropriate, randomized to TA-TAVR or SAVR (control). Nonsurgical candidates were stratified into Cohort B and randomized to TF-TAVI or medical management including ballon valvuloplasty (control, in approximately 80%), whereas patients who did not meet the criteria for TF delivery were excluded from the study because TA implantation was considered too risky. Of the 3,105 patients screened, a total of 1,057 patients (34%) were enrolled at 25 sites in 2 arms—699 patients in Cohort A and 358 patients in Cohort B. All patients were followed up at 30 days, 6 months, and 1 year; and yearly thereafter [24]. For study design scheme, see (Fig. **2**) (adapted from [24]). 

Reynolds *et al*. on behalf of the PARTNER investigators recently reported on the HrQoL outcomes of patients randomized to cohort B. TAVR patients showed significant improvements in the 6-minute walk performance compared with baseline (p=0.002) whereas no improvements were documented in standard therapy patients (p=0.67). In addition, TAVR patients were less symptomatic, had reduced length of hospital stay, and improved physical functioning compared with standard therapy. HrQoL was assessed at baseline and at 1, 6, and 12 months by applying the disease-specific KCCQ and the SF-12. The extent of improvement was large for both disease-specific and generic HrQoL assessment tools and was consistent across all pre-specified subgroups. Significant HRQoL within and between-group improvements were detectable as early as 1 month after TAVR. Whereas KCCQ summary score improved from baseline in both groups, the extent of improvement was greater after TAVR compared with control at 1 month (mean between-group difference, 13 points; p<0.001) with larger benefits at 6 months (mean difference, 21 points; p<0.001) and 12 months (mean difference, 26 points; p<0.001) corresponding to an average improvement of two levels of NYHA class. At 12 months after entry, patients who underwent TAVR had a KCCQ summary score that averaged 25 points higher than the score of those on medical therapy. More than 77% of patients in the TAVI group as compared with 34% in the standard-therapy group derived benefits from baseline of 10 points or greater at 12 months and 62% vs 23% improved by 20 points or more, respectively. At 12 months, TAVR patients reported higher SF-12 PCS and MCS scores with mean differences of 5.7 and 6.4 points, respectively, when compared with standard treatment (p<0.001 for both comparisons) [41].

**Fig. (4):**
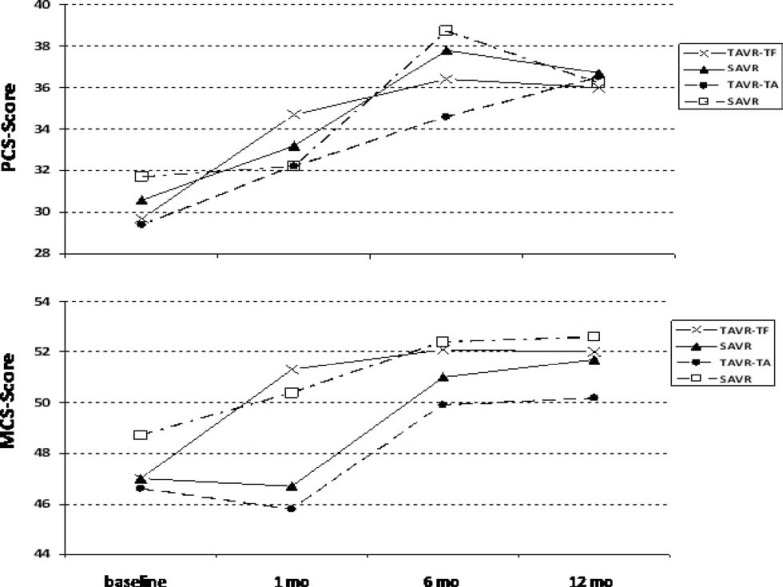
SF-12 PCS and MCS scores from the US PARTNER trial.

Analogously to cohort B, quality of life was assessed using the KCCQ, the SF-12 health status survey and the EQ5D, upon enrollment and at follow-up intervals of 1, 6 and 12 months. Over the 12 months follow-up period both SAVR and TAVR resulted in substantial improvements as quantified by disease-specific KCCQ scale and the generic HrQoL SF-12. Notably, benefits were greater at earlier time points in the transfemoral TAVR group, but became equivalent at 1 year. Whereas in patients after transfemoral access, TAVR resulted in substantial quality-of-life benefits when compared to SAVR at 1 month, benefits at later time points were similar. Interestingly, for patients eligible only for the TA approach, there was no benefit of TAVR over SAVR at any time point, and quality-of-life measures tended to be better with surgical AVR at both 1 and 6 months [42] (Fig. **4**). 

### HrQoL Results from Prospective Single-center Series

Multiple prospective single-center studies added important pieces of evidence to the field confirming that TAVR results in significant health status benefits that are detectable as early as 30 days after valve implantation and detectable up to two years.

In keeping with their previous study reporting mid-term HrQoL improvements after 5 months documenting Significant improvements, Ussia *et al*. recently reported quality-of-life results in their patients at 1-year follow-up. Mean SF-12 PCS scores showed significant improvements from 28.3 to 44.0 at five months and 42.4 at 12 months (p<0.001). Of note, NYHA functional class had improved in all patients. MCS increased from 38.0 to 47.3 at five months and 48.2 at 12 months (p<0.001). Both PCS and MCS in post-TAVR patients were not significantly different from the anticipated thresholds of the general Italian population over the age of 75 years [43, 44]. After previously having demonstrated significant short-term effects after 30 days, Gotzmann and his colleagues were able to show durable improvements at 1 year follow-up as well. By using the MLHFQ and implementing the standardized 6-minute walking test, the HrQoL status and exercise capacity was assessed in 51 patients 30 days and 1 year after TAVR. One year after the procedure HrQoL was significantly improved (baseline score 39.6 ± 19 vs. 26.1 ± 18, p <0.001) as was distance covered in the 6-minute walking test (baseline 185 ± 106 vs 266 ± 118 m, p <0.001). Functional improvements were paralleled by a significant drop of B-type natriuretic peptide levels (baseline 642 ± 634 vs 323 ± 266 pg/ml, p<0.001) and a reduction in left ventricular mass index (156 ± 45 vs 130 ± 42 g/m(2), p <0.001), whereas left ventricular diameter and ejection fraction remained unchanged [45, 46]. 

In a prospective analysis at our own center including 186 patients we could show significant improvements in patients' HrQoL in the short term after 3 months which are maintained up to one year. At 1 year, significant improvements in the SF-36 scores for physical functioning (baseline 34.6 ± 2.3 vs 1 year of follow-up 45.6 ± 2.7; p <0.001), role physical (20 ± 3.0 vs 34.2 ± 4.4; p <0.001), bodily pain (59.9 ± 3 vs 70 ± 2.7; p <0.01), general health (47.3 ± 1.5 vs 55.2 ± 2.1, p <0.001), vitality (35.9 ± 2 vs 48.5 ± 2; p <0.001), and mental health (62.2 ± 2.2 vs 67.3 ± 1.8; p <0.05) were observed when compared to baseline. However, no significant improvement could be detected for social functioning (75.4 ± 2.5 vs 76.5 ± 2.6; p = 0.79) and role emotional (61.1 ± 4.3 vs 66.5 ± 4.7; p = 0.29). At 1 year of follow-up, the various physical and mental scores were comparable to an age-matched standard population. 

Noteworthy, although not statistically significant, the NYHA functional class increased slightly between 3 months and 1 year post-TAVR, with a larger fraction of patients in NYHA class III at 1 year after TAVI (14.1% vs 25.8%). Additionally we could show a high level of patient’s degree of independence and a high proportion of willingness to undergo TAVR again if they would have to decide anew. Although the mental subscales improved slightly, the MCS failed to reach statistical significance in our study population. Higher preprocedural MCS might have limited the sensitivity of the SF-36 questionnaire to detect further postprocedural improvements in mental health. On the other hand, the follow-up period of 12 months may was too short to translate into an improvement of mental health scores. Mental health scores prior to the procedure may be impaired by physical symptoms, psychological problems, adverse treatment effects and social limitations. These factors may lead individuals to withdraw from activities and previous social contacts losing their social relations and social support over a longer period of time preoperatively when cardiac-related symptoms arebecoming more and more apparent. Postprocedurely, it might take longer to reverse the decay of social activities and relationships represented in the SF-36 domains social functioning and role emotional [47, 48]. 

Bekeredjian *et al*. prospectively 87 studied patients out of which 80 survived for 6 months by using the SF-36 health survey. The average scores of all 8 health components had improved significantly after TAVR. The greatest gain was seen in physical functioning (improvement from 23.4 ± 6.0 to 67.8 ± 13.7; p <0.001), whereas lowest gain was seen in bodily pain (improved from 37.5 ± 9.4 to 51.3 ± 11.5; p <0.05). Similarly, both the physical and the mental component summary scores improved significantly. This was consistent with a significant drop in brain natriuretic peptide levels (5,770 ± 8,016 to 1,641 ± 3,650 ng/L; p <0.0001) [49]. 

The PARTNER EU trial prospectively evaluated the procedural and mid-term outcomes of transfemoral or transapical implantation of the Edwards SAPIEN valve. In both groups, 78.1 and 84.8% of patients experienced significant improvements NYHA class, whereas 73.9 and 72.7% had improved KCCQ scores in TA and TF cohorts, respectively [50]. Goncalves *et al*. reported an significant improvement of the NYHA class (2.9±0.4 to 1.4±0.7; p<0.001) which was consistent with an improvement of MLHFQ scores [overall (37.0±14.7 vs. 14.4±10.1; p<0.001), physical dimension (23.2±9.5 vs. 8.6±5.9; p<0.001) and emotional dimension (5.4±4.2 vs. 2.6±3.0; p<0.001)] were significantly improved 6.5months after TAVR [51]. By administering the SF-36 tool and the shorter SF-12 questionnaires in 36 patients before and 1-year after TAVR, Georgiadou *et al*., in keeping with previous studies, could show a significant improvement in both PCS and MCS 1 year after TAVI (baseline vs. 1-year: 21.6 vs 46.7, P < 0.001; 42.9 vs 55.2, P < .001; 22 vs 48.9, P < 0.001; 43.3 vs 52.2, P < .001, respectively) paralleled by a significant change in New York Heart Association class (3 ± 0.7 vs 1.2 ± 0.4, P < .001) [52].

In a total of 102 patients undergoing TAVR, Fairbairne *et al*. evaluated HrQoL by means of two generic health questionnaires (SF-12, EQ-5D) at baseline, 30 days, 6 months, and 1 year according to the recommendations of the Valve Academic Research Consortium HrQoL significantly improved over 1 year (PCS p <0.02; EQ-5D p < 0.02), becoming comparable to age-adjusted US population norms. The greatest change was observed from baseline to 30 days (p < 0.001), with further significant improvements to 6 months (p < 0.01). However, an insignificant decline occurred between 6 months and 1 year (p > 0.05), but a linear pattern of change for PCS and EQ-5D remained (p < 0.05) [53].

Recently, an interesting study was published regarding the comparative HrQoL outcome in high surgical risk patients undergoing either TA-TAVR or SAVR. The SF-36 mental health metascore was similar in both groups (65.6 ± 19 vs. 68.8 ± 22, P = 0.29), while a significant difference was observed in the physical health metascore (49.7 ± 21 vs. 62.0 ± 21, P = 0.015). However, after adjustment for baseline characteristics this difference disappeared indicating that both procedures mediate comparable health status benefits [54].

The recent study Taramasso *et al*. represents the only reporting HrQoL outcomes after TAVR with a follow-up up to two years. In 100 consecutive patients, HrQoL was evaluated with the SF-36 and the MLHFQ at baseline and at two years’ follow-up. Mean SF36-PCS improved from 31.9±8.8 to 51.5±9.5 (p<0.0001); SF36-MCS improved from 44.7±11.6 to 49.5±8.6 (p=0.0002). Mean overall MLHFQ score decreased from 41.5±14.5 to 15.9±13.7 (p<0.0001) [55].

Stortecky *et al*. recently reported that in 62 patients undergoing TAVR HrQoL improved significantly in all components of physical and mental health at nine months: physical functioning (37.0 to 59.0, p<0.0001), physical role functioning (18.3 to 49.1, p<0.0001), general health (55.9 to 64.9, p=0.001), vitality (40.7 to 51.3, p<0.001), social functioning (67.4 to 76.8, p=0.049), emotional functioning (52.0 to 75.8, p<0.001) and mental health (66.6 to 75.8, p=0.05). The subscale bodily pain (60.7 to 70.4, p=0.058) showed a strong trend to improvement, but failed to reach statistical significance. These changes were paralleled by improved NYHA functional class (2.6±0.8 to 1.4±0.6, p<0.0001) [56].

Except for the recent study by Taramasso *et al*., the results of the studies published so far encompass only a follow-up period of a maximum of 1 year, which may be too short. As long as the valve function is durable, one would expect this benefit to be maintained in the longer term as well, but available data suggests that functional and physical health status is slightly worsening again at 12 months. Therefore, the longer term dynamics of HrQoL changes in TAVR patients remain elusive and to be investigated. Another shortcoming in the design of these studies has to be taken into consideration: studies are biased due to deaths or patients who failed to complete the questionnaires at the follow-up period resulting in a study cohort, largely composed by patients who mainly benefited from TAVR.

## 5. PATIENT- AND PROCEDURE-RELATED FACTORS PREDICTIVE FOR QUALITY-OF-LIFE OUTCOMES

While available data among HrQoL “responders” appears promising, the extent to which functional recovery following TAVR is related to patient versus procedural characteristics remains poorly understood and our ability to accurately identify patients who will most likely derive a functional benefit from TAVR is limited. According to the PARTNER trial, an estimated 30% of 30-day TAVR survivors either die or remain highly symptomatic by one year [2]. Obviously, the identification of patient- or procedure-related variables predictive for the extent of HrQoL benefits would be highly desirable in order to facilitate better risk stratification and patient selection, to provide reliable and accurate information for patients, and to finally improve guidance of patient-centered clinical decision making and thus crucial for imporving the overall success of TAVR. Immanent questions are: are symptoms primarily due to the targeted disease process and its sequela (i.e., aortic valve obstruction), are the deleterious effects of the targeted disease process expected to be reversible, and, are there insurmountable obstacles for experiencing HrQoL benefits due to the burden of extensive non-cardiac comorbdidy? On the other hand, even in appropriately selected patients, procedure-realted complications such as vascular or neurologic insults as well residual paravalvular insufficiency may limit functional recovery. 

In the context of the different studies investigating HrQoL after TAVI, both patient characteristics and procedural complications have been described to influence postprocedural recovery, however, predictive factors for the extent of HrQoL changes identified by available studies are variable and incosistent. In Cohort B of the PARTNER Trial the extent of benefit was inferior for patients with oxygen-dependent chronic obstructive pulmonary disease at 6 months. However, at 1-year this association was not significant anymore [41]. Goncalves *et al*. showed that patients with peripheral vascular disease had less benefit in the extent of HrQoL improvement as shown by a lower enhancement in MLHFQ physical dimension score [51]. Interestingly, in a multivariate analysis Fairbairne *et al*. showed that operator´s experience is a predictor of health outcomes in 3 of 4 health surveys, independent of baseline patient characteristics (age, sex, and comorbidities) and procedural complications. Moreover female gender and vascular complications were identified to be independent predictors of lower HrQoL improvements at 1 year [53]. In contrast, procedure-related multiple small cerebral infarcts occurring in 77% of their patients were not associated with an altered health status [57]. In a prospective study performed at our center involving 106 patients completing a 1-year follow-up a mitral valve regurgitation degree of greater than mild was predictive of lower HrQoL improvements. Only, at 3 months this difference reached statistical significance. Likewise, in accordance with Fairbairne *et al*., female gender was also associated with less HrQoL improvements at 3 months However, the difference failed to reach statistical significance at 12 months. Notably, no association could be found for STS- or logEuroScore, other hemodynamic parameters or comorbidities [48].

Although Taramasso *et al*. observed no association between either patient demographics or baseline comorbidities and the degree of post-TAVR functional improvement, residual moderate to severe paravalvular leak and periprocedural stroke were each associated with less substantial improvements in the SF-36 PCS. Moreover, patients with either moderate to severe paravalvular regurgitation or a prolonged length of stay of more than 9 days showed less impressive health benefits in the MLHFQ, although this association was not significant. 

Patients with preoperative chronic renal failure (defined as a serum creatinine >2.0 mg/dl) and obesity (defined as a BMI >30 kg/m2), in spite of a significant improvement of HrQoL, had lower SF-36 PCS at follow-up if compared to patients without these comorbidities (p=0.0009 and p=0.03, respectively). Patients treated via TA delivery route revealed significantly higher MLHFQ scores than those treated by TF or transaxillary approaches, respectively (31.5±32.3 vs. 13.8±8.5 vs. 19.6±11.7; p=0.0006) [55].

In the analysis by Stortecky *et al.*, a lack of health status improvement was present in 32% of all patients and an additional 19% of patients had a minor decrease in HrQoL parameters. Lower or absent improvements were more prevalent among those having experienced a periprocedural complication. However, this difference did not reach statistical significance. Of note, in a multivariable regression model, one study identified the preoperative risk assessment STS score was a predictor for postoperative HrQoL. Amonn *et al*. evinced that every added point in the STS score decreased the SF-36 Physical Health dimension by two raw points at follow-up assessment (P = 0.007) [54]. 

In summary, various predictive factors for the extent of HrQoL changes have been described, but there is still considerable inconsistency. These factors have to be validated in larger studies, in order to serve a patient selection criterion and aid in the decision-making process for the individual patient. A clear definition of comorbid and procedural factors that adversely affect health status benefits despite successful valve implantation is crucial so that this therapy is appropriately used in patients likely to benefit (utility) as opposed to those unlikely to benefit despite successful therapy (futility). For these reasons, HrQoL assessments should continue to be an important component of future TAVR-related trials. 

## 6. TAVR AND COST-EFFECTIVENESS CONSIDERATIONS

The increasing life expectancy and the availability of expensive cutting edge technologies have led to an overproportional escalation of health care expenditures. This trend has put a lot more attention on cost-effectiveness and affordability. TAVR represents an expensive procedure will therefore, as one of many health technologies, compete for funding from a limited healthcare budget. In this respect, beyond the meaningfulness for each individual patient, HrQoL results also have fundamental implications regarding cost and reimbursement deliberations and as a cost-utility tool. Therefore, HrQoL outcomes are an important aspect to the allocation of healthcare resources and a further penetration of the TAVR technology [58]. As to that, cost-utility measures are most commonly used as quality-of-life adjustments to life expectancy in the calculation of quality-adjusted life years (QALYs), which are, in turn, used in clinical decision analyses and cost estimation models. The QALY index has been developed in an attempt to integrate length of life and the degree of health-related quality-of-life improvement into a single metric parameter and is used as a measure of the performance of medical treatments or interventions. The primary outcome of a cost-utility analysis is the cost per QALY, or incremental cost-effectiveness ratio (ICER), which is calculated as the difference in the expected cost of two interventions, divided by the difference in the expected QALYs produced by the two interventions. The QALY index can then be incorporated with medical costs to calculate the cost/QALY ratio which can be used to compare the cost-effectiveness of any treatment. The CER provides the cost for gaining 1 additional QALY and is commonly used to judge whether a treatment is worth its costs compared with an alternative treatment. An intervention with a lower cost to QALY saved (incremental cost effectiveness) ratio ("ICER") is then preferred over an intervention with a higher ratio [59]. 

A few studies investigating cost-effectiveness with respect to TAVR have been published so far. By projecting survival data and costs beyond the trial period in the cohort of inoperable patients, the PARTNER trial added important piece of evidence to the medico-economic evaluation of TAVR. Quality of life was assessed directly from patients at baseline, 1, 6, and 12 months using the EQ-5D health status instrument and were converted to an utility score using a published algorithm developed for the U.S. population. Mean baseline EQ-5D utility scores were 0.59 in the TAVR group and 0.57 in the control group. These increased to 0.71 at 30 days and 0.72 at 6 and 12 months in the TAVR group. Among surviving patients in the control group, EQ-5D scores also increased to 0.64 at 30 days, 0.66 at 6 months, and 0.62 at one year. The differences in utility weights between groups were statistically significant (p<0.05) at each follow-up time point. As a result, when compared to controls, patients undergoing TAVR experience a 278% increase in their projected lifetime QALYs and a 232% increase in their life expectancy. For patients treated with TAVI, mean costs for the initial procedure and hospitalization were calculated to be $42,806 and $78,542, respectively. Follow-up costs through 12 months were lower with TAVR ($29,289 vs. $53,621) due to a mean of 1.2 fewer hospital admissions; however, cumulative 1-year costs remained higher ($106,076 vs. $53,621). On the basis of trial-based survival and cost-projections, the authors projected that over a patient’s lifetime, TAVR would increase discounted life expectancy by 1.6 years (1.3 QALYs) at an incremental cost of $79,837. The estimated higher lifetime costs of patients assigned to TAVI turned out to be almost exactly the same as the cost of the initial TAVI procedure. Due to the patient´s longer life-expectancy, lower costs per year of follow-up were almost exactly counter-balanced by increased medical costs during follow-up [60]. As stated by Reynolds *et al*. and Hlatky *et al*., therefore, the cost-effectiveness of TAVI for patients with inoperable AVS is well within the range of other commonly used cardiovascular technologies. Hlatky *et al*. have put the numbers into a real context for the US: While there is no absolute level of the cost-effectiveness ratio that indicates an acceptable value in the United States, interventions that cost less than $50,000 per QALY added are readily accepted, whereas interventions that cost more than $100,000 per QALY added are generally considered to be too expensive. Interventions between $50,000 and $100,000 per QALY are in an intermediate zone, but are accepted more often than not [60, 61]. 

Based on the health-related quality of life and mortality data from the US PARTNER clinical trial (cohort B) Watt *et al*. projected the cost-effectiveness of the TAVI procedure over a 10-year time horizon in comparison to medical management in patients with severe AVR who are ineligible for conventional SAVR by using a probabilistic decision analytical model. The base case ICER was approximately £16 100 per QALY gained. At a cost-effectiveness threshold of £20 000 per QALY gained independent to changes in key clinical parameters as well as choice of baseline survival data. The observed PARTNER survival data only have to be extrapolated for 2 years to generate an ICER below £30 000 per QALY gained, which is the upper value of the threshold range used by the National Institute for Health and Clinical Excellence in the UK [58]. 

Similarly, in a recent study performed by Gada *et al*., that was based on a decision-analytic model using registry data of high surgical risk TAVR (n=747) and SAVR (n=1199) patients, both TAVR and SAVR turned out to cost-effective when compared to medical management. Post-TAVR utility was assessed on the basis of EQ-5D measures in the EU PARTNER trial. In the reference case, the utility of TAVR was greater than that of SAVR (1.78 vs 1.72 QUALYs). The lifetime cost of TAVR exceeded that of SAVR ($59,503 vs $56,339). The ICER was $52,773/QUALY. Threshold analyses showed that variation in the probabilities of perioperative and annual mortality after SAVR and annual stroke rates post-TAVR were important determinants of the favored strategy [62]. A recent report from Belgium points out that the economic benefit of TAVR is restricted only to “inoperable” patients [63, 64].

Taken together, pertinent analysis suggests that TAVR is highly likely to be a cost-effective treatment modality for patients with severe AVS currently ineligible for SAVR. TAVR satisfies current metrics of cost-effectiveness relative to SAVR and might provide net health benefits at acceptable cost for selected high-risk patients pointing at the need of proper patient selection.

## 7. CONCLUSION AND FUTURE DIRECTIONS

Several registries have conclusively demonstrated safety, efficacy with good clinical results in the short- and long-term. As a result, TAVR has quickly become the standard of care for the treatment of appropriately selected individuals with inoperable AVS. Typically, patients currently referred for and treated by TAVR are elderly with a concomitant variable spectrum of multiple comorbidities, disabilities and limited life expectancy. Quality of life is therefore a key patient-centered outcome. As per current evidence, TAVR in patients with severe AVS ineligible for SAVR alleviates symptoms and is associated with marked functional and HrQoL benefits with sustained effects up to two years when compared with optimal medical care and comparable benefits relative to SAVR. Significant HRQoL improvements are detectable as early a 1 month post-TAVR followed by clinical stabilisation and detectable up to one year. The 1-year health status of TAVR population has been consistently shown to become similar to age-matched general population norms. Calculated on the basis of HrQoL outcomes cost-effectiveness analyzes demonstrate that TAVR is highly likely to be a cost-effective treatment modality for properly selected patients with severe AVS currently ineligible for SAVR. 

Although the understanding of the impact of TAVR on patient´s quality of life is evolving, there are still some questions remaining elusive. Appropriate patient selection will ultimately improve the overall success of the TAVR procedure. Larger patient numbers in conjunction with longer follow-ups will be necessary in order to identify reliable patient- and procedure-related factors predictive for the extent of HRQOL benefits and to answer the question whether benefits are durable conclusively. Especially the impact of the access route via which TAVR is carried out and the influence of procedure-related complications such as stroke, need for permanent pacemaker implantation, vascular complications, residual paravalvular aortic regurgitation or patient-prosthesis mismatch on HrQoL outcomes has to be scrutinized. Insights into treatment outcome of patients with bioprosthetic valve failure (valve-in-valve), bicuspid disease, low-gradient/low-output aortic stenosis, so far not included in randomized clinical trials.In this respects, the role of registries will be of fundamental importance [65]. Randomized controlled trials will add valuable information as to the question how TAVR compares to SAVR. The randomized Surgical Replacement and Transcatheter Aortic Valve Implantation (SURTAVI) study, will enroll 2,500 patients in 75 clinical sites around the world in order to determine if the CoreValve performs better, worse or the same as conventional, open heart surgical techniques [66]. In the time to come, it will be of interest in how far HrQoL benefits compare between TAVR and SAVR in lower surgical risk patients [67]. HrQoL assessments should therefore continue to be an important component of future TAVR-related trials. 

## Figures and Tables

**Fig. (3):**
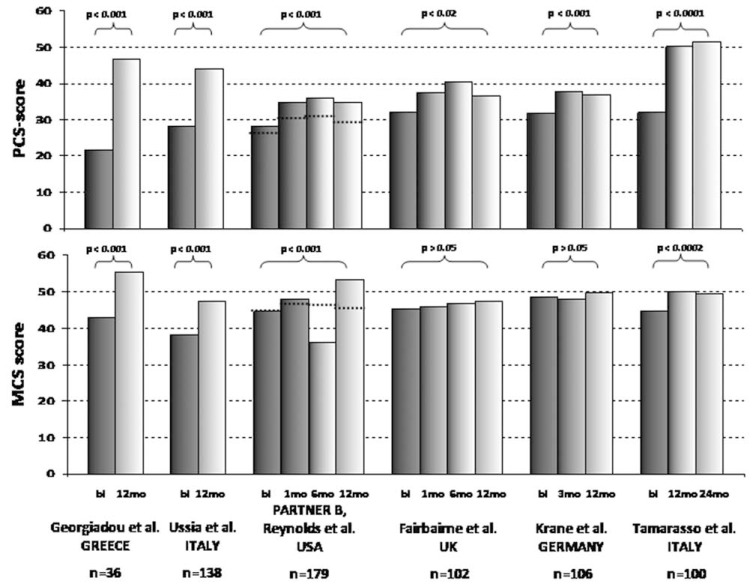
Overview of SF-36 PCS and MCS for prospective studies evaluating HrQoL up to 1 and 2 years, respectively.

**Table 1.** Design and HrQoL outcomes reported in TAVR-related studies.

Study/Year/ Reference	Number of pts. [n]		Mean age [years ± SD]	Mean logEuroScore [% ± SD]	Follow-up [months ± SD]	HrQoL Instrument	Control Group	Valve/Access Site	Main Findings
Ussia *et al*. 2009, [43]		57/30	81.7 ± 4.7	25.3 ± 8.1	5	SF-12	no	Medtronic CoreValve/TF	Mean NYHA: 2.7±0.6 to 1.8±0.5 (p<0.001); Improved (p<0.001) PCS and MCS, return to population norms, greatest change in PF
Gotzmann *et al*. 2010, [45]		44	79.1 ± 7	18.3 ± 12.4	1	MLHFQ	no	Medtronic CoreValve/TF, SC	NYHA III/IV: 90% vs. 16% (p<0.001); 25%increase in 6-minute walk time (p<0.005); significant improvement of MLHFQ overall score 44± 19.1 vs. 28±17.5 (p <0.001); NTpro:BNP: 725 ± 837 pg/ml vs. 423±320 pg/ml (p<0.005)
Krane *et al*. 2010, [47]		99/86	81 ± 6	20	3	SF-36	no	Edwards SAPIEN/TA Medtronic CoreValve/TF	Mean NYHA: 3.1±0.25 vs. 1.7±0.62 (p<0.001); More class I/II at 3 months (NYHA functional class III/IV from 98%to 2% at 3 months) Improved physical health and vitality at 3 months (all p<0.01). No change mental health.
Bekeredjian *et al*. 2010, [49]		87/80	86 ± 2.9	24 ± 15.1	6	SF-36	no	Medtronic CoreValve/TF	Mean NYHA: 3.1±0.5 vs. 1.9±0.6 (p<0.0001); Significant improvements in all 8 health components, PCS and MCS, greatest change in PF (190% increase); NTpro-BNP: 5,8 ± 8,0 ng/L vs. 1,6 ± 3,7 ng/L p < 0.0001).
LefÈvre *et al*. EU PARTNER 2011, [50]		130/107	82.1 ± 5.5	30.0 ± 13.7	1/6/12	KCCQ, EQ-5D	no	Edwards SAPIEN/TF, TA	NYHA III/IV: 84.6% vs. 10.4%,. Of the TF patients, KCCQ score improved in 72.7% and EQ-5D in 51.6%. Among TA patients, KCCQ score improved in 73.9% and EQ-5D in 60.0%.
GonÇalves *et al*. 2011, [51]		74/53	81.6 ± 8	19.3 ± 9.9	6.5	MLHFQ	no	Edwards SAPIEN/TA Medtronic CoreValve/TF	Mean NYHA: 2.9±0.4 to 1.4±0.7 (p<0.001); Significant improvement in MLHFQ scores [overall (37.0±14.7 vs. 14.4±10.1; p<0.001)]
Gotzmann *et al*. 2011, [46]		51	78 ± 6.6	19.6 ± 11.3	1/12	MLHFQ	no	Medtronic CoreValve/TF	NYHA III/IV: 94%, 18%, to 26% (p<0.001); 6MW-Test: 185 ± 106 vs. 248 ± 119 vs. 266 ± 118 (p<0.001); significantly improved MLHFQ score 39.6 ± 19 vs. 26.1 ± 18, p <0.001); NTpro-BNP: 642 ± 634 vs. 323 ± 266 pg/ml (p <0.001)
Georgiadou *et al*. 2011, [52]		36	80.5 ± 5.9	29.7 ± 13.7	11.3 ± 4.9	SF-36, SF-12	no	Medtronic CoreValve/TF, SC	Mean NYHA: 3 ± 0.7 vs 1.2 ± 0.4, (p<0 .001). significant improvement in all domains and summary scale scores, higher than general population norms
Ussia *et al*. 2011, [44]		143/138	81.0±4.6	23.4 ± 14.7	5/12	SF-12	no	Edwards SAPIEN/TF Medtronic CoreValve/TF	NYHA III/IV: 64.3% to 4.2% (p<0.001); marked mid-term improvement in functional status and physical and mental health; PCS 28.3 vs. 44.0 at five vs. 42.4 (p<0.001). MCS 38.0 vs. 47.3 vs. 48.2 (p<0.001).
Reynolds *et al*. PARTNER B 2011, [41]		179	83 ± 9	11.2± 5.8 (STS-Score)	1/6/12	KCCQ, SF-12	Medical n=179	Edwards SAPIEN/TF,TA	Improved 6-MW-Test pre/post at 1 year; no change in no-TAVR group KCCQ; Marked improvement with TAVR at 1 year; improvement in physical and mental HRQOL with TAVR; fewer rehospitalizations at 1 year
Fairbairne *et al*. 2012, [53]		102	80 ± 0.6	20 ± 13	1/6/12	SF-12 EQ-5D, SF-6D	no	Medtronic CoreValve/TF, SC	HRQOL significantly improved over 1 year (SF-12 PCS p = 0.02; EQ-5D p = 0.02; SF-6D p=0.03); similar to age-adjusted U.S. population norms; greatest change from baseline to 30 days (p < 0.001), with further significant improvements to 6 months (p < 0.01).
Amonn *et al*. 2012, [54]		144	79.7 ± 9.2	26.5 ± 16.1	15 ± 10	SF-36	SAVR n=93	Edwards SAPIEN/TA	Similar health metascore in both groups (65.6 ± 19 vs. 68.8 ± 22, P = 0.29), while a significant difference was observed in the physical health metascore (49.7 ± 21 vs. 62.0 ± 21, P = 0.015). After adjustment for baseline characteristics, this difference disappeared.
Krane *et al*. 2012, [48]		186/106	81 ± 6.8	19.74 ± 12.1	3/12	SF-36	no	Edwards SAPIEN/TA Medtronic CoreValve/TF	Mean NYHA: 3.1 vs. 1.9 vs. 2.0 (p<0.001) significant increase in physical scores with a minor change in mental scores, both comparable with age-matched standard population; high degree of independence; 88.6% reaffirmation to undergo TAVR again standard population.
Reynolds *et al*. 2012, [42] PARTNER A		328	83.8 ± 6.8 (TAVR-TF) 82.6 ± 7.0 (TAVR-TA)	11.8 ± 3.2 (STS-Score)	1/6/12	KCCQ, EQ-5D SF-12	SAVR n=216 (TAVR-TF) n=84 (TAVR-TA)	Edwards SAPIEN/TF, TA	Substantial health status improvment between baseline and 1 year after either TAVR or SAVR. TAVR via the transfemoral, but not the transapical route, was associated with a short-term advantage compared with surgery. KCCQ difference TAVR vs. SAVR at 1 month 9.9 (p>0.001).
Taramasso *et al*. 2012, [55]		100	79.7± 6.1	27.9±15.9	12/24	SF-36, MLHFQ	no	Edwards SAPIEN/TF, TA Medtronic CoreValve/TF SC, TAx	Significant improvement in functional status sustained up to two years, 20-point increase in the SF-36 PCS score, 34-point decrease in the MLHFQ
Stortecky *et al*. 2012, [56]		176/62	83±5	22 ± 13	9	SF-36	no	Medtronic CoreValve/TF Edwards SAPIEN/TF	Mean NYHA: 2.6±0.8 to 1.4±0.6 (p<0.0001). improvments in all components of physical and mental health
